# Two‐Layer Droplet Arrays Enable Dynamic Manipulation of Cell Microenvironment During High‐Throughput Bacterial Cultivation

**DOI:** 10.1002/smtd.202501417

**Published:** 2025-10-29

**Authors:** Bijing Xiong, Maximilian Breitfeld, Petra S. Dittrich

**Affiliations:** ^1^ Department of Biosystems Science and Engineering ETH Zurich Schanzenstrasse 44 Basel 4056 Switzerland

**Keywords:** antimicrobial susceptibility testing, droplet manipulation, high‐throughput screening, open microfluidics, persistence, slow‐growing microorganism

## Abstract

Arrays of pico‐to‐microliter droplets, organized on a surface, enable chemical and biological workflows at high throughput. Here, a platform employing two‐layer droplets is presented to enable flexible manipulation of the droplets’ microenvironment for dynamic biological cultivation. Arrays of 6784 agarose droplets (≈2.0 nL per droplet) encapsulating and immobilizing bacterial cells are generated. After that, aqueous droplets (≈3.7 nL) with a defined composition are deposited atop to form a thin liquid layer surrounding the agarose droplets. Chemical exchange between the two layers is extremely fast (equilibrium within 15 s for fluorescein). Moreover, the aqueous layer can be removed, opening the possibility to extract substances from the agarose droplets. Indeed, repeated addition and aspiration of a buffer successfully remove dyes or drugs previously added to the agarose droplets. Therefore, antibiotic drug testing can be performed under both static and transient exposure profiles. The latter reveals that bacterial responses such as bacterial killing and resuscitation are both heterogeneous at the single‐cell level. Last, it is exemplified how such droplet manipulation strategy can also be use in long‐term experimentation, where medium replenishment, performed at 12‐h intervals during a 72‐h experiment, enables the cultivation of a slow‐growing microorganism in nanoliter droplets.

## Graphical abstract

A method is presented to flexibly control the droplet (cell) microenvironment during biological cultivation on droplet microarrays. The method allows for not only highly controlled reagent addition (i.e., single or multistep reagent addition), but also removal of previously added reagents from spatially isolated nanoliter droplets. By doing so, cells can be cultivated and studied in high throughput and under more physiologically relevant conditions.

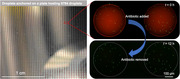

## 1 Introduction

Microdroplet arrays (also referred as droplet microarrays or droplet arrays) consist of thousands of picolitre‐to‐microliter‐sized droplets, typically arranged in a distinct pattern on an open surface. Commonly, droplets are immersed in an inert oil or stored a humid environment to prevent evaporation. Microdroplet arrays allow cell cultivation and analysis to be performed in high throughput and at microscale with unique benefits.^[^ ^1^, ^2^, ^3^, ^4^, ^5^, ^6^ ^]^ Comparing to cell cultivation in closed microfluidic platforms or microdroplets stored in closed systems.^[^ ^7^, ^8^, ^9^, ^10^ ^]^ several attractive features appear when cells are cultivated in thousands of droplet microcosms that are anchored on an open substrate. First, temporal monitoring of cells and their behaviors is feasible throughout the cultivation due to the anchorage of droplets on the substrate surface. Studies on mammalian cells including adherent cells are possible,^[^ ^11^, ^12^ ^]^ as well as different microbial species.^[^ ^2^, ^13^, ^14^ ^]^ Second, droplets on open arrays are accessible–which offers the potential to actively manipulate the droplet/cell microenvironment during cultivation and/or prior cell analysis. Third, the accessibility opens the possibility for further analysis beyond microscopy such as mass spectrometry,^[^ ^3^, ^15^ ^]^ which can be directly performed on‐plate. In addition to that, droplet(s) of interest can be recovered for off‐plate analysis or for further cultivation,^[^ ^16^ ^]^ which is critical especially in directed evolutionary studies and (rare cell) screening assays.

Microdroplet arrays have been used in many different studies, including high‐throughput drug testing and screening.^[^ ^1^, ^2^, ^17^, ^18^ ^]^ Typically, the drug candidates are provided together with the medium and cells at the beginning of the experiment–namely drugs are tested under static conditions. However, in real‐world clinical practices, drugs such as antibiotics are typically applied repeatedly in a cyclic fashion during the course of a treatment. Different outcomes have been observed in pathogens when exposed to such respective (transient) antibiotic therapies.^[^ ^19^, ^20^ ^]^ Hence, the ability to dynamically manipulate droplets microenvironment during biological cultivation will enable us to cultivate and study cells under more physiologically relevant conditions. In addition to that, medium replenishment posts another urgency for cell cultivation when the size of the cultivation vessels is at nanoliter scale. Medium replenishment is inevitable when cultivating cells in nanoliter droplets over a long period (i.e., >72 h) to replenish the required growth factors, amino acids, nutrients and carbon source.^[^ ^21^, ^22^, ^23^ ^]^ In addition, loss of water can occur,^[^ ^7^, ^24^ ^]^ i.e., droplets shrink, despite they are covered by an inert and hydrophobic oil.

To manipulate the microenvironment of droplets on open arrays, several attempts have been made.^[^ ^25^, ^26^ ^]^ For instance, by actuating and merging droplets loaded with different cargos (i.e., cells and reagents (s) of interests, respectively), the target reagent(s) can be delivered to the cells in a stringently controlled fashion.^[^ ^27^, ^28^, ^29^, ^30^ ^]^ One limitation of such droplet fusion is however the throughput, despite efforts have been made to perform the actuation and fusion of multiple, i.e., *n* = 3 droplet pairs at one time.^[^ ^31^ ^]^ In this regard, a promising method for high‐throughput droplet manipulation is sequential liquid deposition–where liquid carrying reagent(s) of interest is deposited subsequently (and repeatedly) atop the cell‐laden droplets pre‐existing on an open array.^[^ ^32^ ^]^ However, disruption of cells and cell cross‐contamination between droplets are common in sequential liquid‐liquid deposition, as cells loaded in the aqueous droplets are planktonic and thus can be easily carried over from one droplet to another. In addition to that, only a limited number of depositions can be performed in sequential liquid deposition as the droplet volume increases with each deposition and thus hindering an active manipulation of the droplet microenvironment. Lastly and importantly, due to the lack of microchannels, current methods only allow for reagent addition but not their removal from the spatially isolated cultivation droplets anchored on the arrays. Reagent removal is however an essential step in creating a dynamic droplet microenvironment and sometimes mandatory prior many bioanalytical analysis.^[^ ^33^ ^]^ Additionally, reagent removal is also needed in cultivations when toxic cellular byproducts are produced.

Recently, we have developed a method for rapid creation of large microdroplet arrays.^[^ ^34^ ^]^ In this method, liquid droplets are created on a substrate that is immersed in an oil bath by shearing off an aqueous stream delivered through a capillary. Here, we adapted this method to create novel two‐layer (hydrogel‐water) microdroplet arrays with over 6700 discrete droplets to enable flexible droplet manipulation for dynamic biological cultivation at high throughout. First, we create hydrogel droplets, where cells of interest are encapsulated and immobilized, and covered under oil to prevent evaporation. Subsequently, a second aqueous droplet with a defined chemical composition is deposited on top of the hydrogel droplet, which allows exchange of compounds loaded in the aqueous droplet into the hydrogel and vice versa. Thereby, the hydrogel droplet and their insoluble cargos remain spatially undisturbed, yet the aqueous layer acts as a chemical reservoir which can not only be deposited but also aspirated in a highly repeatable fashion. In this way, single, repetitive, or multistep addition of compounds to the cells and the removal of previously added compounds from the hydrogel droplets can be realized in a high‐throughput fashion. Meanwhile, cells immobilized within the hydrogel droplets can be easily observed and tracked by time‐lapse microscopy. In this study, we demonstrate the use of the platform in highly parallel bacteria cultivation, drug testing under both static and transient antibiotic exposures. Additionally, we also demonstrate long‐term cultivation enabled by medium replenishment at defined frequencies to achieve cultivation of a relatively slow‐growing microorganism in nanoliter microdroplets in high throughput.

## 2 Results and Discussion

### 2.1 Formation of Agarose‐Water Two‐Layer Droplet Arrays

**Figure 1:**
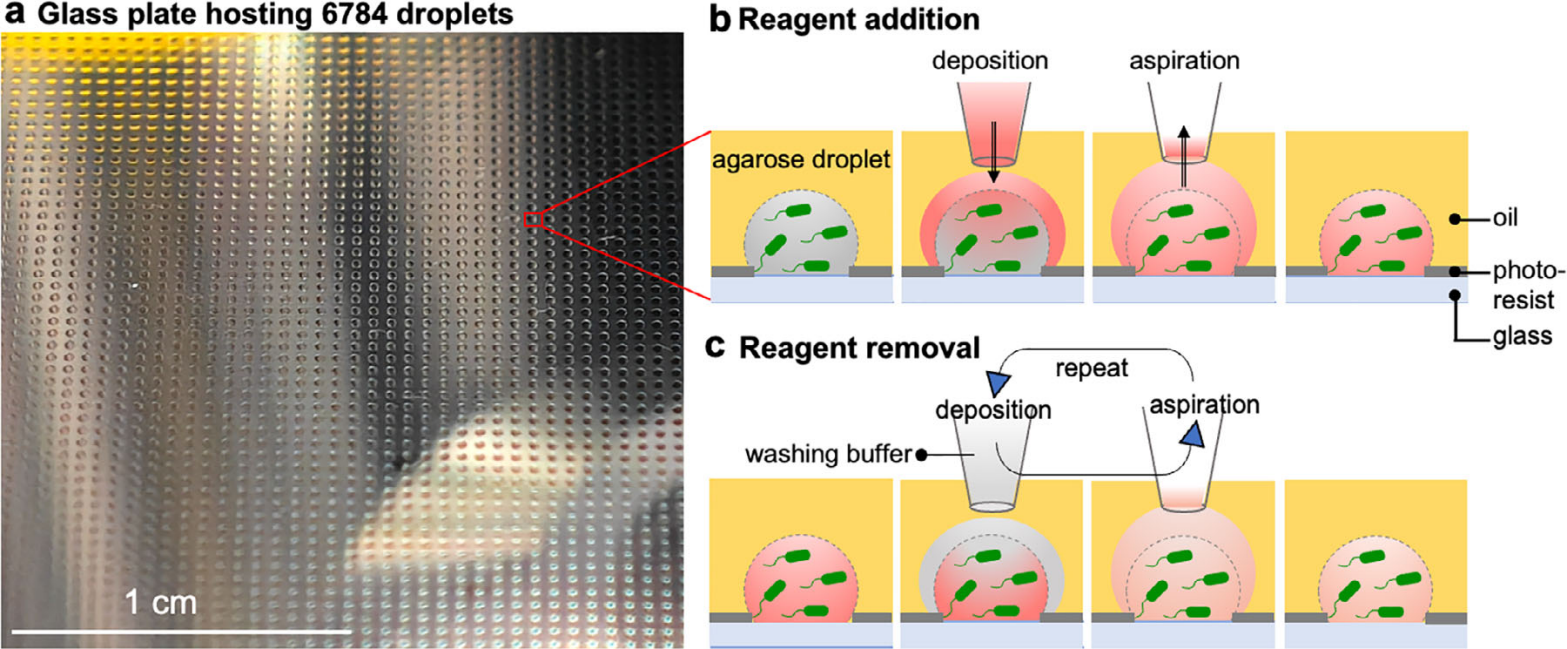
Agarose‐water two‐layer droplets on open arrays for dynamic control of the droplet microenvironment. a) A sectional photograph of agarose droplets anchored on the droplet array. b and c) Sketches (side view), illustrating reagent addition b) and reagent removal c), respectively. A capillary is placed just above the agarose droplet to deliver the aqueous layer or aspirate it. At the same time, the glass plate is moved underneath, and the fluid shears off to form the droplet (process depicted in Video S1, Supporting Information). Repeated deposition and aspiration cycles as shown in c lead to gradual reagent dilution and eventually reagent removal.

Our platform consists of a glass substrate covered by a hydrophobic layer embedding *n* = 6784 hydrophilic spots to anchor the hydrogel (**Figure** **1**a) and aqueous droplets. The glass plate is placed in an oil bath and mounted on a motorized microscope stage. The hydrogel or aqueous phase is delivered through a capillary, whose nozzle end is positioned closely above the glass substrate. When moving the glass substrate underneath, droplets are formed on the hydrophilic spots. This process of rapid microdroplet array creation by shear streaming has been described recently.^[^ ^34^ ^]^ Here, we deposit first hydrogel droplets to encapsulate and immobilize the otherwise planktonic bacterial cells. After gelation, an aqueous phase with a defined chemical composition can be deposited atop the agarose droplets to add (Figure 1b) or to remove (extract) (Figure 1c) the reagent(s) of interest in the agarose droplets. For these purposes, we selected an agarose with a low‐gelling temperature (*T* < 35 °C) to form the hydrogel droplet layer, as it allows for not only a facile deposition of hydrogel droplets at a cell‐compatible temperature, i.e., *T* = 37 °C, but also the subsequent deposition of the second aqueous layer (Video S1, Supporting Information) due to its hydrophilic surface property.^[^ ^35^ ^]^ The latter is of particular importance as some polymers’ surface properties change significantly before and after the polymerization. For instance, fibrinogen and thrombin are often used to form fibrin hydrogel (at 37 °C or lower temperature).^[^ ^36^ ^]^ However, during the transformation the polymer's surface properties change from very hydrophilic (fibrinogen) to hydrophobic (fibrin).^[^ ^37^ ^]^ The hydrophobic fibrin gel droplets are water‐repellent and thus, it was not possible to deposit the second aqueous layer atop the fibrin droplets (Video S2 and Figure S1, Supporting Information). Apart from the surface properties, volumes of the droplets are also controlled, and they are dependent on the viscosity of the target solution, the flow rate in the capillary, the moving speed of the microscope stage underneath and the deposition height of the capillary. A systematic study had been previously performed to characterize the impact of these parameters on the droplets volume and described elsewhere.^[^ ^26^ ^]^ During the two‐layer droplets generation, the parameters were selected to rapidly generate agarose and water droplets with an average volume of 2 and 3.7 nL, respectively. Such droplet volumes allow for an efficient droplet manipulation whilst without causing droplets merging or incomplete liquid removal due to excessive volume (i.e., v > 4 nL) during the droplet manipulation. Lastly, when applying a negative pressure in the capillary atop the agarose‐water droplets, positioned ≈40 µm above the aqueous layer, the liquid layer can be removed (Video S3 and Figure S2, Supporting Information). The distance of 40 µm was selected so that the liquid layer could be efficiently removed whilst the bottom agarose layer would not be impacted. Simultaneous aspiration of water and agarose droplets occurred occasionally when the capillary was operated at ≈20 µm distance (Figure S3, Supporting Information), which could be useful under the context of recovering droplets of interests. Whereas aspiration at a far distance such as ≈60 µm resulted in incomplete liquid aspiration (Figure S3, Supporting Information). With parameters described in the SI, agarose‐water two‐layer droplets allow rapid chemical exchange between the two phases, whilst the second layer of liquid (namely the chemical reservoirs) can be reliably deposited and aspirated in a highly repeatable fashion, yet the agarose droplets and their insoluble cargos such as cells remain spatially undisturbed. Empowered by our advanced liquid handling platform, one aqueous deposition or aspiration on the droplet array hosting *n* = 6784 agarose droplets can be done within 10 min and thereby, enabling flexible droplet manipulation at high throughput.

### 2.2 Reagent Addition

**Figure 2:**
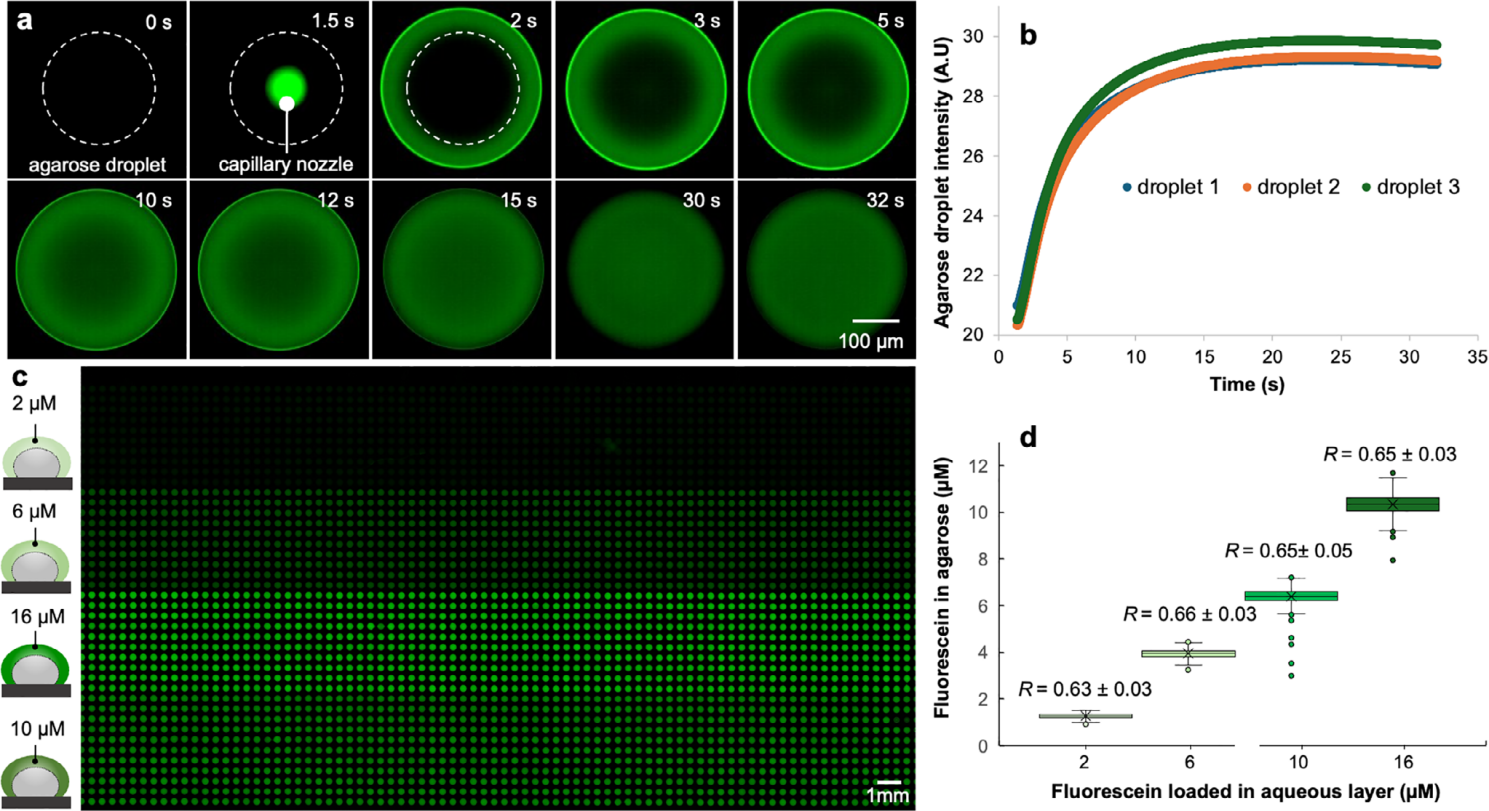
Visualization of reagent addition. a) High‐speed imaging (200 frames per second) shows the deposition of a fluorescein‐laden aqueous layer atop an agarose droplet (indicated by the dashed circle) and the rapid diffusion of fluorescein into the agarose droplet until equilibrium. b) Time‐lapse analysis of the fluorescence intensity in the agarose droplet after the deposition of the fluorescein‐laden aqueous layer at *t* = 2 s (n = 3 agarose‐water pairs). c) A micrograph of agarose droplets after the deposition of aqueous layers containing fluorescein at different concentrations. The liquid layer was removed 2 min after the deposition and images of the agarose droplets were taken after that. d) Reagent addition rate (*R*_addition_) calculated by dividing the agarose fluorescein concentration with the initial fluorescein concentration loaded in the aqueous layer in c). The fluorescein concentration in the agarose droplets was estimated using a pre‐established calibration curve (Figure S4, Supporting Information) correlating fluorescein concentration and its intensity in agarose droplets, *n* =1280 droplets per concentration, and *n* = 1408 droplets for fluorescein addition with 10 µm initial concentration. No significant difference (*p* > 0.05, one‐way ANOVA) was observed in the *R*_addition_ when the agarose droplets were treated with liquid containing fluorescein at different concentrations.

Next, we systematically characterized reagent addition in agarose‐water two‐layer droplet arrays. We deposited a solution with fluorescein (5 mM) atop the agarose droplets and observed instant fluorescein diffusion into the agarose droplet after the aqueous deposition with a high‐speed camera (**Figure** **2**a and *t* = 2–15 s). Equilibrium between the agarose and aqueous layer was reached within 15 s (Figure 2b). Mathematically, using Fick's first law of diffusion, a maximal equilibrium time of 18.3 s was also predicted for the two‐layer droplets (see simulation details in Supporting Information). This rapid chemical exchange is explicitly enabled by the small volumes of both the liquid layer and the agarose droplets. After a rapid chemical exchange was confirmed, we next quantitatively measured the final concentrations achieved in the agarose droplets when manipulated with aqueous solutions carrying the target reagent (i.e., fluorescein, Figure 2c) at different concentrations.

As the volume of both the agarose and aqueous layer was controlled and known (i.e., 2.0 and 3.7 nL per agarose and water droplet, respectively, see calculation in SI), a reagent addition rate *R*_addition_ = (*C*_agarose_)/(*C_0_*_aqueous_) = 0.65 can be calculated for the system after the equilibrium. Experimentally, a *R*_addition_ = 0.65 ± 0.03 was measured (*n* = 5248 droplets for four different fluorescein addition concentrations, Figure 2c,d). Noteworthy, the recipient agarose droplets had a homogenous fluorescence intensity if received fluorescein addition from the same solution (concentration), indicating homogeneous reagent addition and a maximal standard deviation of the final fluorescein concentration of 4.5% was observed in agarose droplets received 10‐µm aqueous fluorescein addition (Figure 2d). Moreover, when the target reagent is loaded in the liquid layer, a concentration gradient can also be automatically generated within the agarose droplets (i.e., Figure S5, Supporting Information) by depositing the liquid layer with a gradient pressure. Additionally, neither microbial nor chemical cross‐contamination were observed during the liquid deposition and/or aspiration (Figures S6 and S7, Supporting Information). These results show that despite operating at nanoliter scale and at high throughput, our reagent addition is rapid, homogeneous and quantitative. Yet it is important to note that despite the pore sizes of agarose typically range from 50 to 600 nm (which allows virtually all soluble chemicals to freely diffuse in and out of the agarose droplets), this however may not allow an efficient diffusion of big molecules such as enzymes. Thus applications involving big molecules may require specific characterization on the diffusion of the target reagent in agarose.

### 2.3 Highly Parallel Bacterial Cultivations and Antimicrobial Susceptibility Test

**Figure 3:**
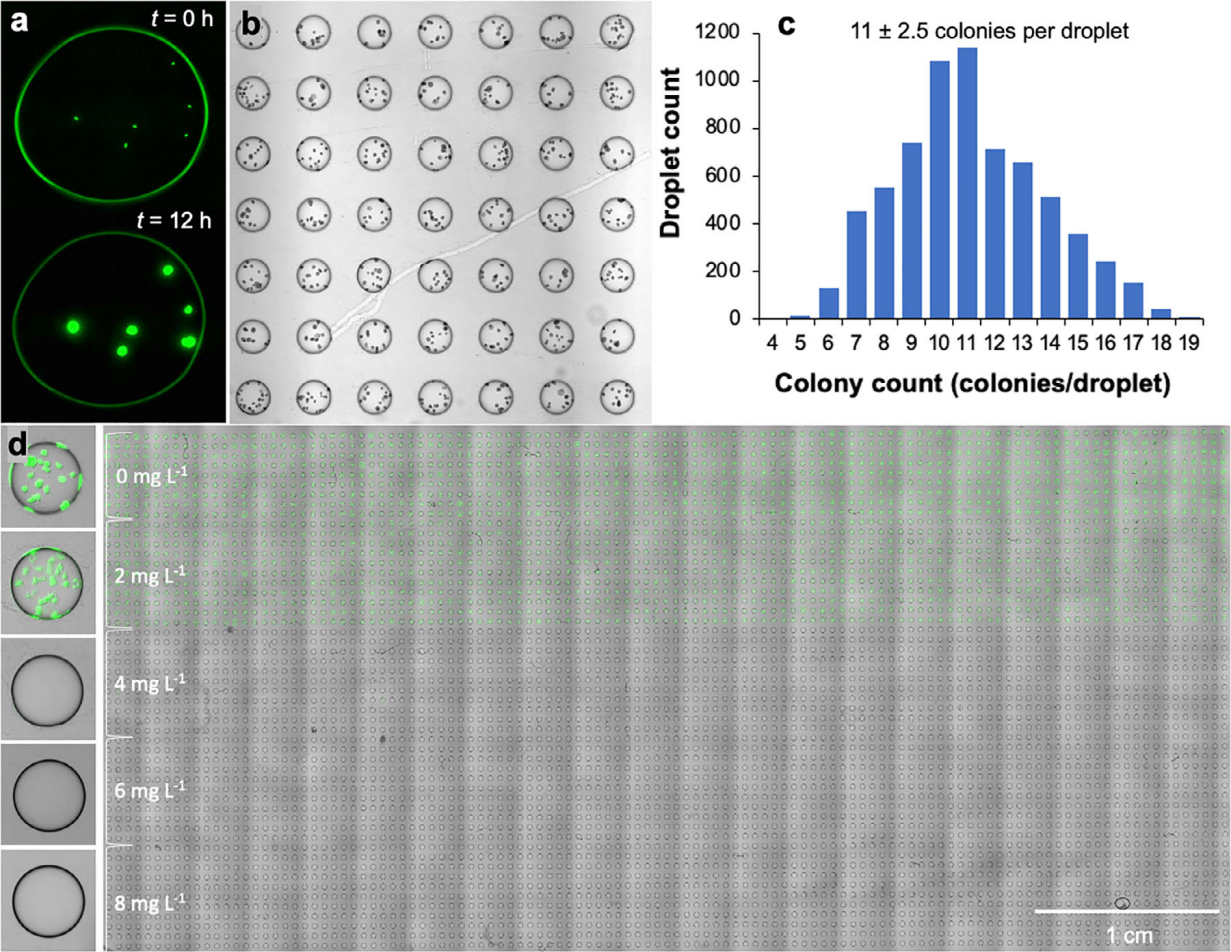
Bacterial growth in agarose droplets and antimicrobial susceptibility testing. a) Fluorescence micrographs show initial bacterial encapsulation (*t* = 0 h) and their subsequent “one viable cell one colony formation” growth principle (*t* = 12 h) in a representative agarose droplet (droplet dimeter: 250 µm). b) Bright field micrograph showing colony formation in agarose droplets after 12 h incubation. c) A histogram illustrating colony formation frequency in agarose droplets *(n* = 6784), initial bacterial OD_600_ was 0.01. d) Growth (colony formation) of *E. coli* ATCC25922 in agarose droplets supplied with ampicillin at different concentrations using the two‐layer droplet reagent addition technique. n = 1152 droplets for 0 mg L^−1^ AMP, and n = 1408 droplets for 2, 4, 6, 8 mg L^−1^ AMP, respectively.

Next, we demonstrate the use of the platform in highly parallel bacterial cultivation for antimicrobial susceptibility test (AST) (**Figure** **3**). First, we encapsulated *E. coli* ATCC25922 cells during agarose droplet formation. Different from bacterial growth in aqueous droplets, where cells replicate and then distribute within the whole droplet, cells encapsulated in the agarose droplets are immobilized and thus one viable cell forms one colony (Figure 3a). We hence could observe the encapsulated cells and later quantify colony‐forming units (cfu) microscopically. For instance, after 12 h incubation, the colonies were clearly visible even by brightfield microscopy and could be easily counted without the need of a fluorescent label (Figure 3b and Video S4, Supporting Information).

In a typical experiment, the OD_600_ of the agarose suspension was adjusted to OD_600_ = 0.01 so that, in average we encapsulated 11 ± 2.5 cells per droplet (Figure 3c) on the *n* = 6784 droplet array. For antimicrobial drug testing, we first generated agarose droplets with *E. coli* ATCC25922 cells, incubated them for 15 min and subsequently added the antibiotic ampicillin (AMP) at different concentrations (final concentration calculated according *R*_addition_ = 0.65, Figure 3d). We determined a minimal inhibitory concentration (MIC) of 4 mg L^−1^ (Figure 3d) which is in accordance with the MIC range reported by the traditional approaches (EUCAST table). This result further reflects reagent addition is highly controlled, and the platform is suitable for applications such as AST, which requires a quantitative manipulation of the droplet microenvironment. Additionally, due to the high throughput trait of the microarrays and the “one viable cell on colony formation” growth principle in agarose, our platform may be a powerful system to quantitatively locate rare event within a bacterial population such as heterogeneous antimicrobial resistance. Heterogeneous antimicrobial resistance is a phenomenon that only a small number of cells in a bacterial population (i.e., one in a million) are resistant to antibiotics which are often overlooked and difficult to quantify with conventional antimicrobial susceptibility test approaches.^[^ ^38^ ^]^

### 2.4 Reagent Removal and Transient Drug Exposure

**Figure 4:**
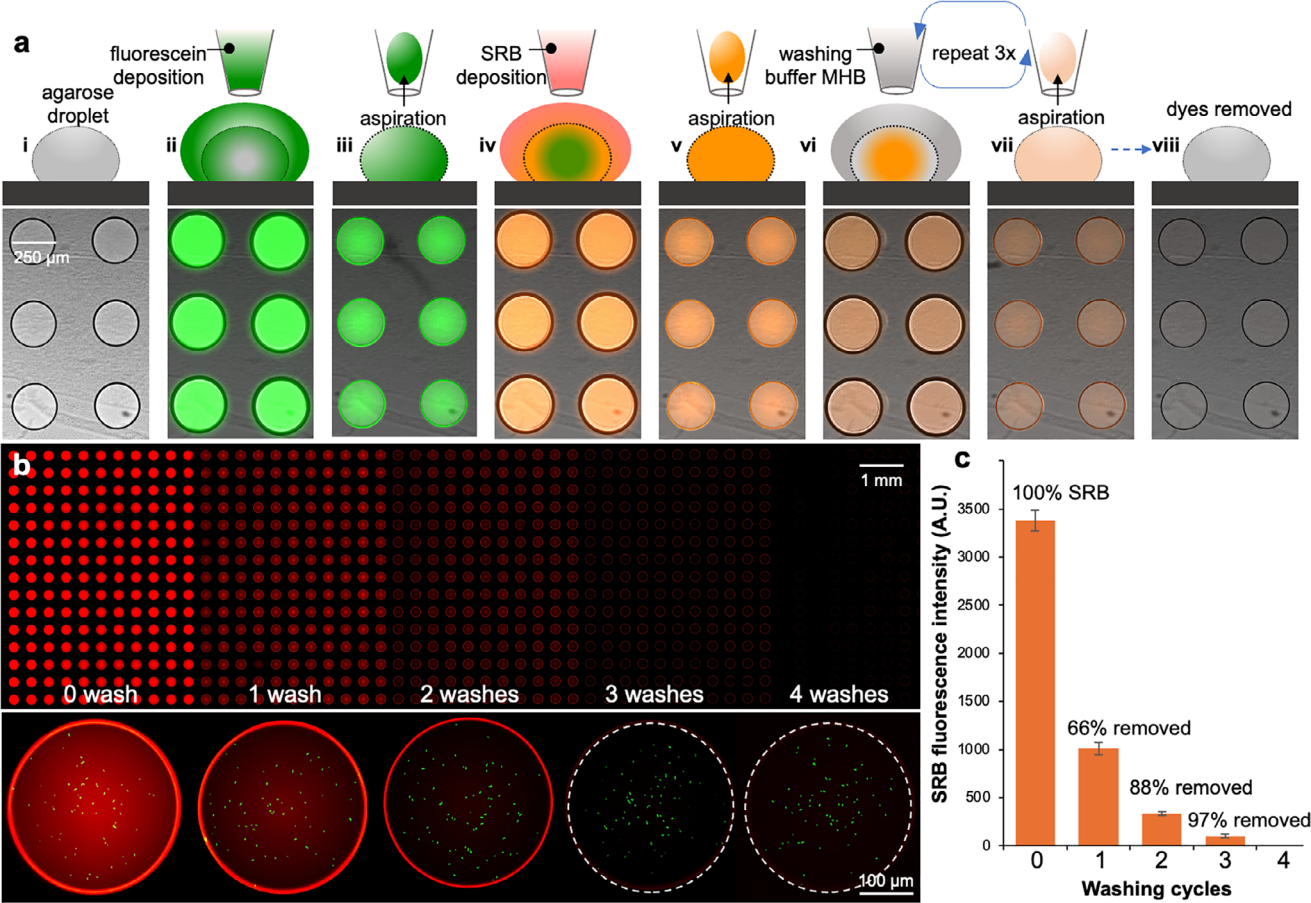
Reagent removal. a) Sketches depicting the workflow and micrographs of a subset of droplets showing the outcome of each operation. i–v) sequential addition of two different fluorescent dyes (fluorescein and then SRB, i–v) and subsequently, their removal vi–viii) from the agarose droplets via repetitive deposition & aspiration of solutions with different chemical compositions. b) Micrographs of SRB‐laden agarose droplets after receiving different times of repetitive deposition & aspiration of the washing buffer MHB (“washing cycles”). Initially, all agarose droplets on the array were loaded with 20 µM SRB, and then droplets were repeatedly “washed” with SRB‐free MHB medium. Note that the GFP‐expressing cells remained in the hydrogel layer regardless of the frequency of washing cycles. c) Quantitative analysis of SRB fluorescence intensity in agarose droplets after different washing cycles. Without washing, the fluorescence intensity detected in the agarose droplets was 3380 ± 103 A.U., and it was decreased to 1014 ± 64, 334 ± 17, and 100 ± 16 A.U. after 1, 2, and 3 washing cycles, respectively. *n* = 1408 droplets per washing cycle, *n* = 1152 for 4 times washing.

In many biology studies, not only reagent addition but also their removal is required, e.g., washing steps involved in transient drug exposure experimentations.^[^ ^39^, ^40^ ^]^ Agarose‐water droplet arrays enable these advanced operations. While the cells are embedded within the hydrogel, the aqueous droplet on top can be removed. Several rounds of deposition and aspiration of the water droplets facilitate the dilution and eventually full removal of compounds from the agarose droplets. We first demonstrate this advanced manipulation with two different fluorescent dyes (5 µM fluorescein and 10 µM sulforhodamine B (SRB)) that were sequentially added to the agarose droplets by the deposition and aspiration of a fluorescein‐laden and then, an SRB‐laden solution, respectively (**Figure** **4**a i–iv). Subsequently, repetitive aspiration and deposition of a “washing buffer” (Mueller Hinton broth, MHB medium) was performed for another three times (Figure 4a v–vii). After that, neither fluorescein nor SRB was detected microscopically (Figure 4a viii). During the addition and then removal of fluorescein and SRB, liquid deposition and aspiration were repeatedly performed 5 times in total on the agarose droplets. Moreover, after the 5‐time droplet manipulation, the agarose droplets remained physically intact (Figure 4a viii).

To quantitatively describe reagent removal using the two‐layer droplets, we next experimentally measured the reagent removal rate (*R*_removal_) with different washing cycles (Figure 4b,c). Agarose droplets initially loaded with 20 µM SRB (molecular weight 558.67 g mol^−1^) were generated together with the GFP‐expressing *E. coli* ATCC25922 cells. After that, an SRB‐free MHB was repetitively deposited and aspirated on the agarose droplets (Figure S8, Supporting Information), respectively. An *R*_removal_ in each droplet was then calculated by dividing the reduced SRB fluorescence intensity with the SRB intensity initially detected in the droplet. As shown in Figure 4b,c, 20‐µM SRB pre‐existing in the agarose droplets was observed to be readily (>97%) removed after three times’ repetitive deposition and aspiration of the washing buffer MHB. The SRB fluorescence signal was too low to be detected after the fourth deposition and aspiration (Figure 4b,c). Noteworthy, the GFP‐expressing cells remained in the hydrogel layer regardless of the amount of washing cycles being performed on the agarose droplets (Figure 4b bottom panel).

**Figure 5:**
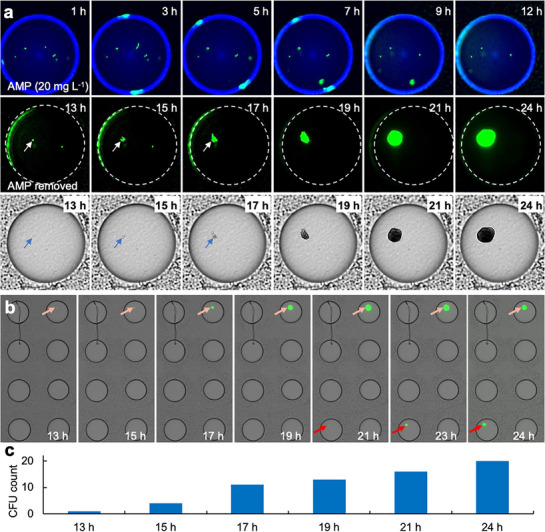
Transient drug exposure. a) Time‐lapse fluorescence and brightfield micrographs of a representative droplet showing bacterial responses to transient AMP exposure (Figure S9, Supporting Information depicts the entire array). At *t* = 1–12 h, in the presence of AMP (indicated by the presence of dextran blue which was co‐administered with AMP), some of the *E. coli* cells in the droplet were lysed and yet three of them remained morphologically intact even after 12 h of AMP treatment (*t* = 12 h). When AMP was removed (as indicated by the disappearance of dextran blue) from the droplet, one of the morphologically intact cells (as pointed by the arrows) started to regrow and formed a colony over time (*t* = 13–24 h). b) Micrographs showing colony formation in another two representative droplets at two different time points after AMP removal. c) Total colony counts in the n = 6784 droplet array at different time points after AMP removal.

This universal approach, where the cell‐laden agarose droplets reliably adhere on the substrate surface whilst the outer aqueous layer can be repeatedly deposited and aspirated, opens the door to create highly dynamic conditions within the agarose droplets during high‐throughput biological cultivation. To exemplify how this feature can be exploited, we performed drug testing under transient antibiotic exposure (**Figure** **5**). In this study, cells of *E. coli* ATCC25922 were encapsulated in the agarose droplets. Afterwards, AMP together with a blue‐fluorescent dye dextran (as an indicator for the presence or absence of AMP in agarose droplets) was added to the agarose droplets and cultivated for 12 h (Figure 5a; see Figure S9, Supporting Information for the whole array). After that, AMP was removed, and the AMP‐removed droplets were further cultivated for another 12 h. In the presence of AMP (*t* = 1–12 h, Figure 5a), the majority of the cells were lysed by AMP and no bacterial growth was observed in any of the 6784 droplets. However, once the AMP was removed from the droplets, some of the bacterial cells that remained morphologically intact resuscitated (*t* = 13–24 h, Figure 5a) and formed observable colonies over time. The regrowth was an extremely rare event, and we found only 20 colonies in total on the entire plate, with ≈70 000 colonies that could be expected if without AMP treatment. Interestingly, by counting the colonies formed on the microarray over time, we further observed that the resuscitation of different *E. coli* cells began at different time points during the antibiotic‐free cultivation (Figure 5b,c). Such heterogeneous responses of bacterial killing and resuscitation in the presence and absence of antimicrobials have also been reported in other studies,^[^ ^41^, ^42^, ^43^, ^44^ ^]^ which enable microorganisms to survive the temporally and spatially highly dynamic environmental conditions typically observed in all microbial habitats.^[^ ^45^ ^]^ In the context of infection treatment, single‐cell heterogeneity can lead to treatment failures of antibiotics.^[^ ^46^, ^47^, ^48^ ^]^ Our approach allows to mimic such highly dynamic environmental conditions in the cultivation vessels and thereby, to perform drug testing under more physiologically relevant conditions.

### 2.5 Medium Replenishment for Long‐Term Cultivation

**Figure 6:**
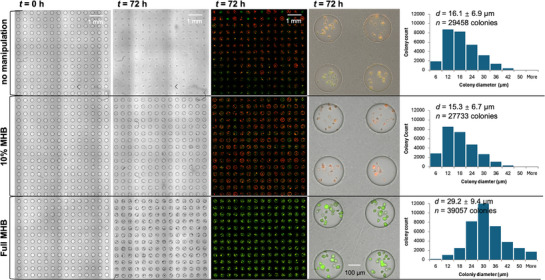
Medium replenishment for cultivation of a relatively slow‐growing bacterium. Brightfield and fluorescence images of an array of droplets encapsulating *M. smegmatis* (Figure. S10, Supporting Information depicts the entire array) that were cultivated in agarose droplet under different conditions for 72 h. On different rows of the array, either no medium replenishment or different medium was replenished at 12 h intervals. The green and red colony color reflect viable and dead cells, respectively. The right histograms characterize the quantity and size of the observed colonies under each condition. The average size of colonies formed in droplets with full MHB replenishment was significantly bigger (*p* < 0.05, one‐way ANOVA) than the size of colonies formed in droplets with 10% MHB replenishment or no replenishment. On the microarray, n = 1920 droplets with no medium replenishment or replenishment with 10% MHB respectively, n = 2944 droplets with full MHB replenishment.

Another limitation of droplet microfluidics is the long‐term cultivation of microbial cells in small volumes, mostly due to the loss of water and depletion of nutrients over time. Our method enables medium replenishment for long‐term cultivations, which we demonstrate with a relatively slow‐growing microorganism (*Mycobacterium smegmatis*, GFP‐expressing, doubling time 3–4 h). We first encapsulated *M. smegmatis* in MHB agarose droplets and then cultivated the microdroplet arrays at *T* = 37 °C for 72 h. During the cultivation, droplets at different rows received different manipulations (**Figure** **6**). Without any manipulation (upper rows in Figure 6; Figure S10, Supporting Information), droplets shrunk and even dried out almost completely after 72 h. Consequently, only a small amount of bacterial biomass was observed in these droplets with an average colony diameter of 16.1 ± 6.9 µm. Moreover, most of the colonies in these droplets (>95% of the *n* = 29 458 colonies) were not viable as indicated by the dead cell stain propidium iodide (PI). In contrast, when the medium was replenished with full MHB medium (rows at the bottom of Figure 6 and row 32‐52 in Figure S10, Supporting Information) in 12 h intervals, *M. smegmatis* was observed to grow and formed GFP‐expressing colonies (an indicator of mature and functioning colonies.^[^ ^49^, ^50^ ^]^) with an average diameter of 29.2 ± 6.4 µm after cultivation for 72 h. Notably, only few colonies (<1% of the *n* = 39 057 colonies) were stained by PI in these droplets. For comparison, we also supplied a tenfold‐diluted MHB to the agarose droplets also at a frequency of 12 h to mimic a water‐replenished yet nutrient‐deprived condition (middle rows in Figure 6). Nutrient deprivation/competition is a common factor typically observed to inhibit the cultivation and isolation of slow‐growing organisms in the presence of fast‐growing organisms. In such condition, *M. smegmatis* formed significantly smaller colonies (*d* = 15.3 ± 6.7 µm, *p* < 0.001), which were mostly not viable after 72 h, as > 97% of the *n* = 27 733 colonies were stained by PI. (Figure 6) In summary, the active droplet manipulation enabled by the two‐layer microdroplet arrays also applies in long‐term experiments and we could realize cultivation of the relatively slow‐growing *M. smegmatis* for 72 h in nanoliter droplets. With such medium replenishment technique, it also becomes possible to co‐cultivate microorganisms that are difficult to be co‐cultivated with conventional approaches where microbial competition on nutrients and carbon sources is the limiting factor of their co‐cultivation.^[^ ^51^ ^]^

## 3 Conclusion

In this study, we formed two‐layer droplet arrays, consisting of an agarose droplet layer serving as cultivation shells, and an aqueous droplet layer serving as chemical reservoirs to allow flexible yet high‐throughput droplet manipulation for dynamic biological cultivation. We demonstrated rapid and highly controlled reagent addition as well as reagent removal via serial dilutions from spatially isolated nanoliter droplets. We exemplified how the parallelized microbial cultivations and the highly controlled droplet manipulation can be used for drug testing. Importantly, while drug tests are commonly done under static conditions, our method enables the transient exposure of antibiotic drug in high throughput, thereby mimicking the dosing profile in patients’ body. This advanced option opens the way to reveal time‐dependent cell response to drugs which may drive cells to develop antimicrobial persistence or resistance. Such droplet manipulation can also be used for medium replenishment at defined frequencies and thereby, enabling long‐term cultivation of a relatively slow‐growing microorganism in nanoliter droplets. In addition to these applications, we envision the reagent removal methodology reported here also offers solutions to several challenges typically associated with cell cultivation and analysis in high‐throughput droplet microfluidics. For example, reagent removal makes it possible to remove biologically active reagents or to remove the endogenously produced toxic catalysts, or for cell processing prior analysis. In this context, the shrinkage of droplets over several days can be further controlled and reduced by proper encasing of the platform in a gas‐tight frame. Beyond our demonstrations, we anticipate this robust droplet manipulation strategy would enormously increase the achievable complexity of both the experimental and analytical settings on high‐throughput droplet arrays.

## 4 Experimental Section

### Fabrication of the Droplet Array Plates

The droplet array plate was patterned with *n* = 6784 hydrophilic spots (Ø = 250 µm) organized in a format of 53 rows by 128 columns, where both the row and column spacing were set to 200 µm, respectively. The fabrication process of the plates was adapted from.^[^ ^34^ ^]^ In brief, a 600 nm layer of polysilazane (CAG37, durXtreme, Germany) was spin‐coated onto a 380 µm fused silica glass wafer (Siegert Wafer, Germany) at 1000 RPM for 30 s. Subsequently, the wafer was placed on a hotplate for 10 min at 300 °C. Next, a 70 µM layer of negative resist (AZ nLoF 2070, Micro resist technology, Germany) was spin coated at 3000 RPM for 30 s followed by a soft bake for 90 s at 110 °C. The wafer was covered by a photomask (Selba, Switzerland), and the array design was transferred onto the wafer by UV with an exposure dose of 1500 mJ cm^−2^ (MABA8‐Gen3 mask aligner, Karl Suss, Germany). The wafer was developed with AZ 726 MIF (Micro resist technology, Germany) for ≈120 s to remove the unpolymerized resist. The pattern given by the photolithography process was transferred into the polysilazane layer by reactive ion etching (RIE 100, Oxford Instruments Plasma Technology, UK) followed by a 120‐s rinse in buffered hydrofluoric acid (6:1, Sigma Aldrich, USA). Afterward the resist covering the polysilazane was stripped‐off with acetone (Sigma Aldrich, USA), followed by an iso‐propanol (Sigma Aldrich, USA) and then water rinse. Finally, the plate was cut out from the wafer with a dimension of 75 × 35 mm.

### Microorganisms, Medium and Growth Conditions

Two model organisms*, E. coli* ATCC25922 (GFP‐expressing.^[^ ^52^ ^]^) and *Mycobacterium smegmatis* (*M. smegmatis*, GFP‐expressing, a kind gift from Prof. John McKinney, EPFL) were used in this study for different purposes (see below). Monocultures of the two strains were cultivated in a cation‐adjusted MHB medium (Sigma Aldrich, USA) at 37 °C in a shaking incubator with a shaking speed of 200 rpm. Additionally, kanamycin (Sigma Aldrich, USA) was supplied to *E. coli* ATCC295522 at a concentration of 100 µg mL^−1^ for the plasmid maintenance during the cultivation. After an overnight cultivation (48‐h cultivation for *M. smegmatis*), monocultures of the two strains were harvested, then their OD_600_ measured and both adjusted to OD_600_ = 0.1, equivalent to a cell density of ≈8 × 10^7^ cells mL^−1^.^[^ ^53^ ^]^ The harvested culture from *E. coli* ATCC25922 was used for the antimicrobial susceptibility test experiment (see Section Reagent Addition) as well as drug testing under transient ampicillin exposure experiment (see Section Drug Testing Under Transient Ampicillin Exposure). Whereas the monoculture of *M. smegmatis* was used in the long‐term cultivation experiment (see Section Medium Replenishment During Long‐Term Cultivation).

### Microscopic Imaging and Data Analysis

For microscopic imaging, a fully automated microscope (Eclipse Ti2, Nikon, Japan) equipped with LED light source, objectives (Plan Apo 4x and 10x) and a complementary metal oxide semiconductor (CMOS) camera installed within an environmental chamber were used. Different filter sets including GFP, mCherry and DAPI were used to record signals from the GFP‐labeled cells (or fluorescein), PI and dextran, respectively. Microscopic images were analyzed using ImageJ.

### Generation of Agarose‐Water Two‐Layer Droplet Arrays

The platform for droplet generation was described elsewhere.^[^ ^34^ ^]^ In brief, droplets are generated by shearing off a stream of the target solution that is delivered within a pressure‐controlled capillary (IDEX, USA, inner diameter = 100 µm) via moving the plate with a high‐precision stage (U‐781 PILine, Physik Instrumente, Germany). Different parameters were used for depositing agarose droplets and the aqueous phase carrying the reagent of interest, which is described in an additionally supplemented protocol (SI). In brief, first 20‐µL bacterial inoculum with an OD_600_ = 0.1 was added to 180‐µL 1.1% agarose (Agarose Low Melt ROTIGarose, Carl Roth, Germany) to achieve a final OD_600_ = 0.01 and a final agarose concentration of 1% for bacterial encapsulation during the formation of agarose droplets (step 1 in the supplemented SI protocol). After that, the tray carrying the agarose droplets (in HFE oil, HFE‐7500 3 m, Novec, USA) was taken out of the environmental chamber and cooled down at room temperature for the gelation of the agarose droplets (gelling temperature <35 °C). After that, an aqueous phase carrying the reagent(s) of interest were deposited atop the agarose droplets following step 2 in the protocol (SI). Once a chemical equilibrium was achieved between the aqueous layer and the agarose droplets, the aqueous layer could be aspirated by applying a negative pressure to the capillary following step 3 in the protocol (SI). Of note, the droplet array was kept within the environmental chamber at 37 °C after the gelation in all further droplet manipulation and cell cultivation processes (see below). Noteworthy, the melting temperature of the agarose is 65 °C thus the agarose droplets can remain their solid phase after the gelation despite being kept at 37 °C.

### Reagent Addition

Agarose droplets (1% agarose in MHB) were generated on the droplet array. After that, an aqueous phase carrying 5‐mM fluorescein (Sigma Aldrich, USA) loaded in MHB medium was deposited atop the agarose droplets. During the aqueous deposition, a high‐speed camera (MIRO M110, Phantom, USA) was used to image the disposition process, as well as the subsequent diffusion and equilibrium of fluorescein from the aqueous layer to the agarose droplets. The high‐speed camera was set to take fluorescent images of the droplets at a frequency of 200 frames per second (fps) for 32 s.

After that, a calibration experiment was performed to quantitatively describe reagent addition in the two‐layer droplets system. First, agarose droplets containing different concentrations of fluorescein between 0 and 12 µm were generated at different rows of the *n* = 6784 droplets array to establish a calibration curve (Figure. S4, Supporting Information). Secondly, another droplet array was used to generate (i) agarose droplets (fluorescein free), and then (ii) a liquid carrying fluorescein at a concentration between 0 and 16 µm was deposited atop the agarose droplets located at the different rows of the array, respectively. Two min after the fluorescein deposition, the aqueous layer was aspirated. A reagent addition rate (*R*_addition_ = $\frac{c\;(agarose)}{c_{0}\;\left(aqueous\right)}$) in each droplet could be calculated by dividing the final fluorescein concentration detected in the agarose droplets with the fluorescein concentration initially loaded in the outer aqueous layer.

Lastly, to validate the measured *R*_addition_, an antimicrobial susceptibility test (AST) experiment was performed. For this, agarose droplets encapsulating *E. coli* ATCC25922 cells (GFP producing) were first generated on the droplet array. Fifteen min after cell encapsulation, MHB medium containing antibiotic ampicillin (AMP) at a concentration of 0, 3.1, 6.2, 9.3, 12.4 mg L^−1^ was deposited to the agarose droplets located at the 1–9, 10–20, 21–31, 32–42, 43–53 rows of the array respectively. The solutions of different AMP concentrations were prepared before in Eppendorf tubes, and the supplying capillary was inserted in the respective tube during spotting. Two min after the deposition of different AMP‐laden MHB solutions, the aqueous layer was then aspirated. After that, the droplet array (in HFE oil) was sealed with a plastic membrane (Thermo Fisher Scientific, USA) and incubated in the environmental chamber at 37 °C for 12 h.

### Reagent Removal

To quantify the removal of reagents from the agarose droplets, agarose droplets containing the fluorescent dye SRB at a concentration of 20 µm were generated on the droplet array. After that, an SRB‐free MHB solution (the “washing buffer”) was deposited to the agarose droplets and then aspirated 2 min after the deposition. Such aqueous deposition & aspiration (“droplet washing cycle”) were performed for 0, 1, 2, 3, and 4 times on the SRB‐laden agarose droplets located at the 1–10, 11–21, 22–32, 33–43, and 44–53 rows of the array (Figure S8, Supporting Information), respectively. Fluorescent images were taken before and after different “washing” cycles using the Nikon microscope with a 4x objective plus a 1.5× amplification, with an exposure time of 20 ms. Fluorescence intensity of the droplets was then analyzed to calculate the reagent removal rate R_removal_ by dividing the reduced SRB intensity with the initial SRB intensity detected in the droplet.

### Drug Testing Under Transient Ampicillin Exposure

*E. coli* ATCC 25 922 cells were encapsulated in the MHB agarose droplets. Fifteen min after the cell encapsulation, a MHB solution containing AMP (30 mg L^−1^), PI (a dead cell stain, 10 µM) and a blue, fluorescent dye dextran (as an indicator of successful AMP addition or AMP removal, 0.6 mg L^−1^) was deposited and then aspirated atop the cell‐laden agarose droplets to achieve an AMP concentration of 20 mg L^−1^. The droplet array was sealed with a plastic membrane and then cultivated at 37 °C for 12 h in the presence of AMP. After that, a MHB medium containing only PI was repetitively deposited and aspirated atop the agarose droplets for three times to remove the previously added AMP and dextran whilst preserving the PI in the agarose droplets. The AMP‐removed agarose droplet array was then cultivated and monitored microscopically for another 12 h with a 10× objective.

### Medium Replenishment During Long‐Term Cultivation

An agarose droplet array encapsulating a relatively slow‐growing microorganism *M. smegmatis* (GFP‐labelled) was generated. After that, the agarose droplet array (in HFE oil) was sealed with a plastic membrane and incubated in the environmental chamber at 37 °C for 72 h. During the 72‐h cultivation, the plastic membrane sealing the droplet tray was removed whenever droplet’ manipulation was performed. On the *n* = 6784 droplets array, no operations were performed on agarose droplets located at the 1‐16 rows of the array, whereas 10‐time diluted MHB (10% MHB) and full MHB medium were deposited atop agarose droplets located at the 17–31, 32‐53 rows of the array respectively, both at a frequency of 12‐h intervals (thus medium in these droplets was exchanged six times in total). In the last round of droplet manipulation at *t* = 72 h, 10 µM of the dead cell stain PI was included in a PBS solution, the PI‐laden PBS was deposited to all droplets on the array and then aspirated 2 min after the deposition. Microscopic images were taken using the 4× objectives at 12‐h intervals during the 72‐h incubation.

### Statistical Analysis

Microsoft Excel software (version 2025) was used for data processing. SPSS (version 30.0) was used to verify normality of data with Shapiro‐Wilk's test. Means were then compared using one‐way ANOVA followed by either the LSD test or Dunnett's T3 test, depending on equal variances were or were not assumed, respectively, to compare differences between multiple groups.

## Conflict of Interest

The authors declare no conflict of interest.

## Supporting information



Supporting Information

Supplemental Video 1

Supplemental Video 2

Supplemental Video 3

Supplemental Video 4

## Data Availability

The data that support the findings of this study are available from the corresponding author upon reasonable request.
