# Supplementary material for: Two‐Layer Droplet Arrays Enable Dynamic Manipulation of Cell Microenvironment During High‐Throughput Bacterial Cultivation
Source: Small Methods. 2025 Oct 29;9(12):e01417. doi: 10.1002/smtd.202501417 (PMC12716177; doi:10.1002/smtd.202501417)
Supplement: Supplementary file 1 — Supporting Information [file SMTD-9-e01417-s003.pdf]

Supporting Information

Two-Layer Droplet Arrays Enable Dynamic Manipulation of Cell Microenvironment During High-Throughput Bacterial Cultivation

*Bijing Xiong[1], Maximilian Breitfeld[1], and Petra S. Dittrich[1]**

B. J. Xiong, M. Breitfeld, and P. S. Dittrich
[1]Department of Biosystems Science and Engineering, ETH Zurich
Schanzenstrasse 44, 4056 Basel, Switzerland
E-mail: petra.dittrich@bsse.ethz.ch

This supporting material includes:

1. ***A deposition and aspiration protocol to generate and manipulate agarose-water two-layer droplets***
2. ***Ten supporting figures with captions***
3. ***Calculation of the droplets' volume***
4. ***Mathematical simulation of diffusion in agarose-water two-layer droplet system***
5. ***References***
6. ***Description for the supplementary videos***

1. Protocol for the Generation and Manipulation of Agarose-Water Two-Layer Droplets on Open Microarrays

A fully automated droplet deposition platform (1) incorporated within an environmental chamber was used to deposit (or aspirate) the different layers of the two-layer droplets. The temperature within the environmental chamber was constantly kept at 37°C throughout the droplets' generation, manipulation, and cultivation processes. In brief, our droplet generation platform deposits droplets by spotting a continuous flow of the target solution delivered within a pressure-controlled capillary on an array plate via moving the microarray plate with an automated high-precision stage. Below are detailed parameters used to control the deposition/aspiration pressure, stage movement and deposition/aspiration conditions to generate or manipulate the agarose-water two-layer droplets.

All components of the two-layer droplets were deposited or aspirated using a capillary with an inner diameter of 100 µm (IDEX, the US) and a length of 23 cm. To generate the two-layer droplets, the agarose suspension inoculated with the target cells (kept at 37°C) and the aqueous solution carrying the reagent(s) of interests were deposited using parameters described in **Step 1** and **Step 2,** respectively. Whereas for the aspiration of the aqueous layer, the parameters are described in **Step 3**.

Step 1: depositing the cell-laden agarose droplets (~ 2 nL per droplet)

Spotting route: from row 1 to row 53 (can be defined flexibly depending on which rows are wished to be deposited);
Deposition direction: snake-by-rows;
Spotting/stage moving speed: 6 mm/s;
Spotting capillary height: 0.07 mm above the microarray plate;
Spotting pressure: 300 mBar.

After the deposition of the agarose droplets, the capillary was flushed with de-ionized water, ethanol (both kept at 37°C) and then N_2 under a pressure of 1000 mBar for 1 min, respectively. Meanwhile, the tray carrying the agarose droplets (in HFE oil) was taken out of the environmental chamber and cooled down at room temperature for 10 min for the gelation of the agarose droplets (gelling temperature < 35°C). After that, the tray was put back to the stage and an aqueous phase carrying reagent(s) of interests could be deposited atop the agarose droplets following parameters described in **Step 2**.

Step 2: depositing the aqueous layer (~ 3.7 nL per droplet)

Spotting route: from row 1 to row 53 (can be defined flexibly depending on droplets located at which rows are wished to be manipulated);
Deposition direction: snake-by-rows;
Spotting/stage moving speed: 6 mm/s;
Spotting capillary height: 0.11 mm above the microarray plate;
Spotting pressure: 45 mBar.

After the deposition of the aqueous layer, the capillary was flushed with de-ionized water, ethanol (both kept at 37°C) and then N_2 under a pressure of 1000 mBar for 1 min, respectively. After that, a negative pressure was applied to the capillary to aspirate the aqueous layer atop the agarose droplets at a distance of ca. 40 μm above the aqueous layer following parameters described in **Step 3** (see below).

Step 3: aspiration of the aqueous layer

Spotting route: from row 1 to row 53 (can be defined flexibly depending on which rows of water droplets are wished to be aspirated);
Aspiration direction: snake-by-rows;
Spotting/stage moving speed: 6 mm/s;
Spotting capillary height: 0.16 mm above the microarray plate;
Spotting pressure: -700 mBar.

By repeating **Step 2 & 3**, the aqueous layer can be deposited and aspirated in a repeatable fashion atop the agarose droplets. During long-term experiments, i.e., > 72-h droplet cultivation, the height of agarose, or agarose-water two-layer droplets could change over time due to evaporation. Accordingly, the spotting capillary height (both in **Step 2** and **3**) may need to be adapted, and the general principle is that the capillary nozzle should be set at a distance of ~ 40 μm from the agarose or water droplets. In our study, the aforementioned nozzle distance was functioning throughout a 72-h cultivation at 37°C.

2. Supporting Figures

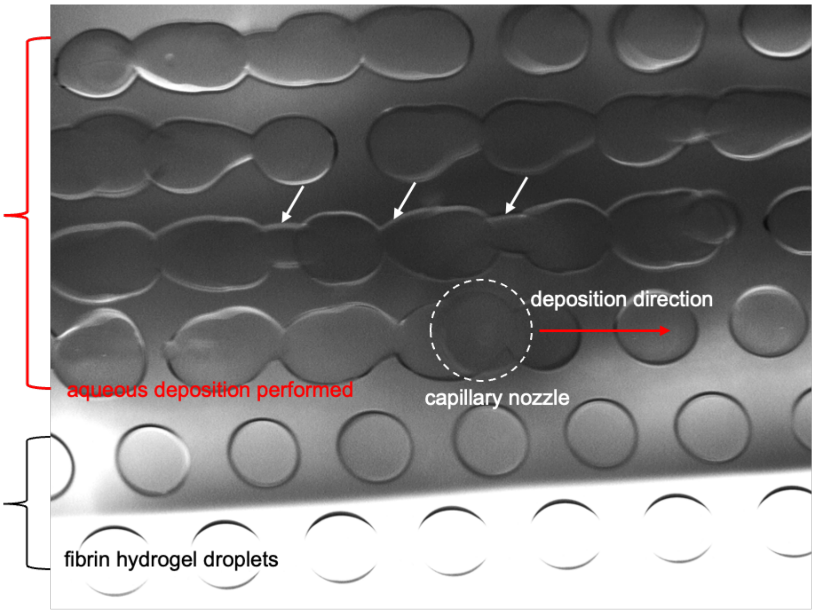

Figure S1. A snapshot of aqueous deposition atop the fibrin droplets. Due to the hydrophobic surface properties of the fibrin (2) droplets, the deposited MHB aqueous droplets were repelled by the fibrin droplets and parts of the liquid was pushed to sit in-between the two neighboring fibrin droplets (as pointed by the white arrows). Hence, instead of forming spatially isolated and two-layered water-fibrin droplets, the aqueous deposition atop the hydrophobic fibrin droplets resulted in the formation of fibrin-water droplet chains, as shown in the upper four rows in the micrograph.

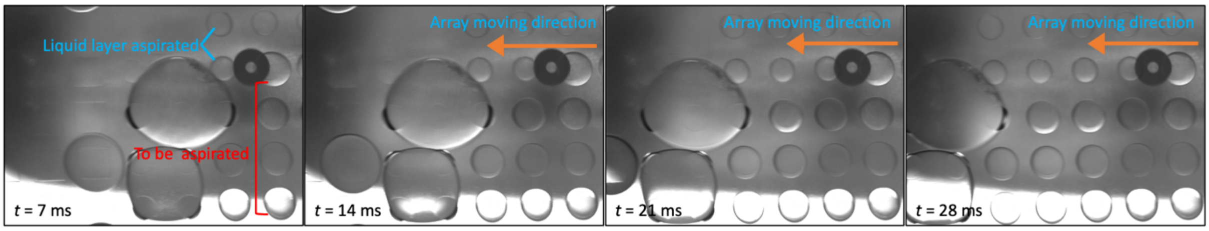

Figure S2. Snapshots (from Video S3) showing liquid aspiration from the agarose-water two-layer droplets. The micrographs were taken at a frequency of 120 frames per second.

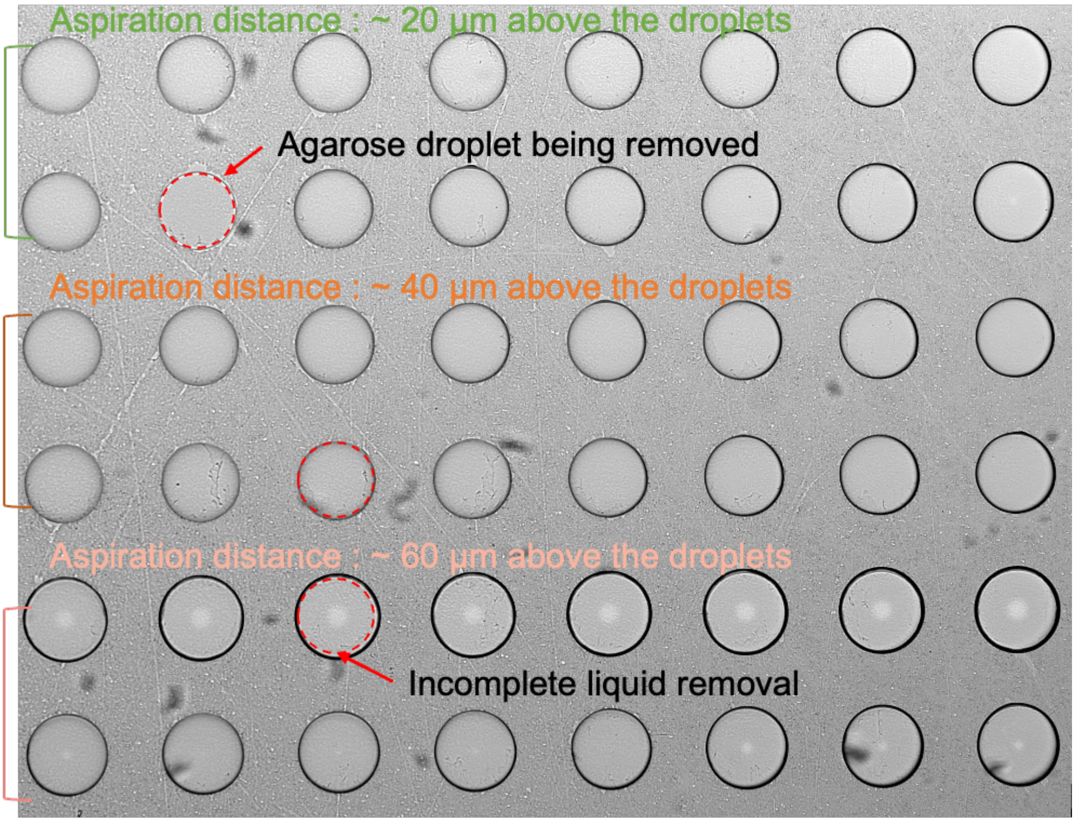

Figure S3. Selection of capillary aspiration height. An aspiration distance of h = ~ 40 μm was selected as it allowed for an efficient liquid removal yet without removing the agarose droplets at the bottom layer.

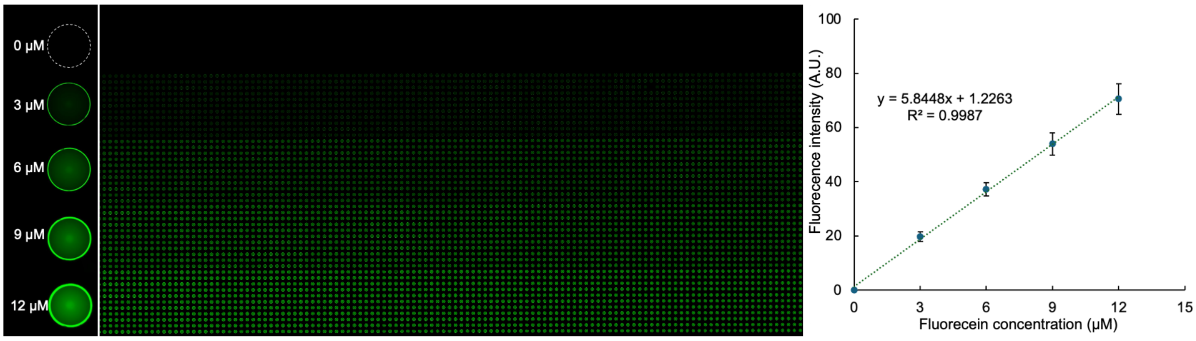

Figure S4. Calibration of fluorescein intensity and its concentration in agarose droplets. Left panel, a fluorescence micrograph of agarose droplets containing different concentrations of fluorescein. **Right panel**, a calibration curve correlating fluorescein fluorescence intensity and its concentration.

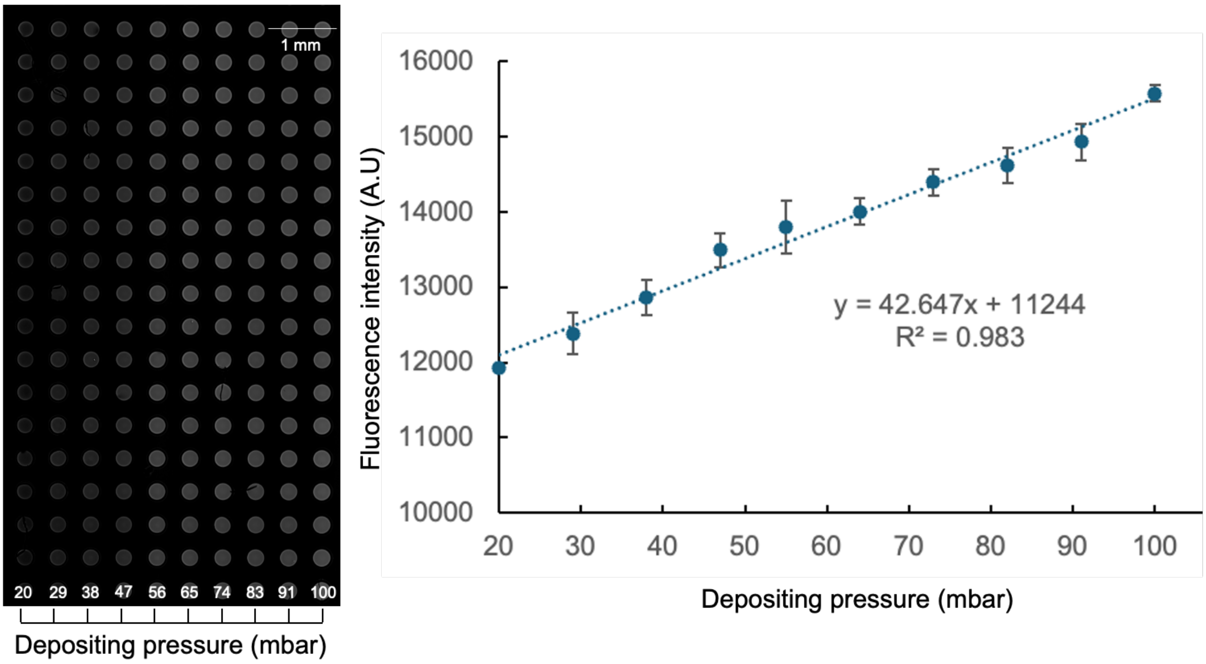

Figure S5. Concentration gradient generation using two-layer droplets microarray. A gradient pressure (from 20 mbar to 100 mbar in a step of 9 mbar) was used to deposit the liquid layer loading 50-μM SRB atop the agarose droplets. The liquid layer was later removed by two-time liquid aspiration with a -700 mbar pressure on all droplets. The SRB intensity detected in the agarose droplet presented a positive linear correlation with the pressure applied for liquid deposition.

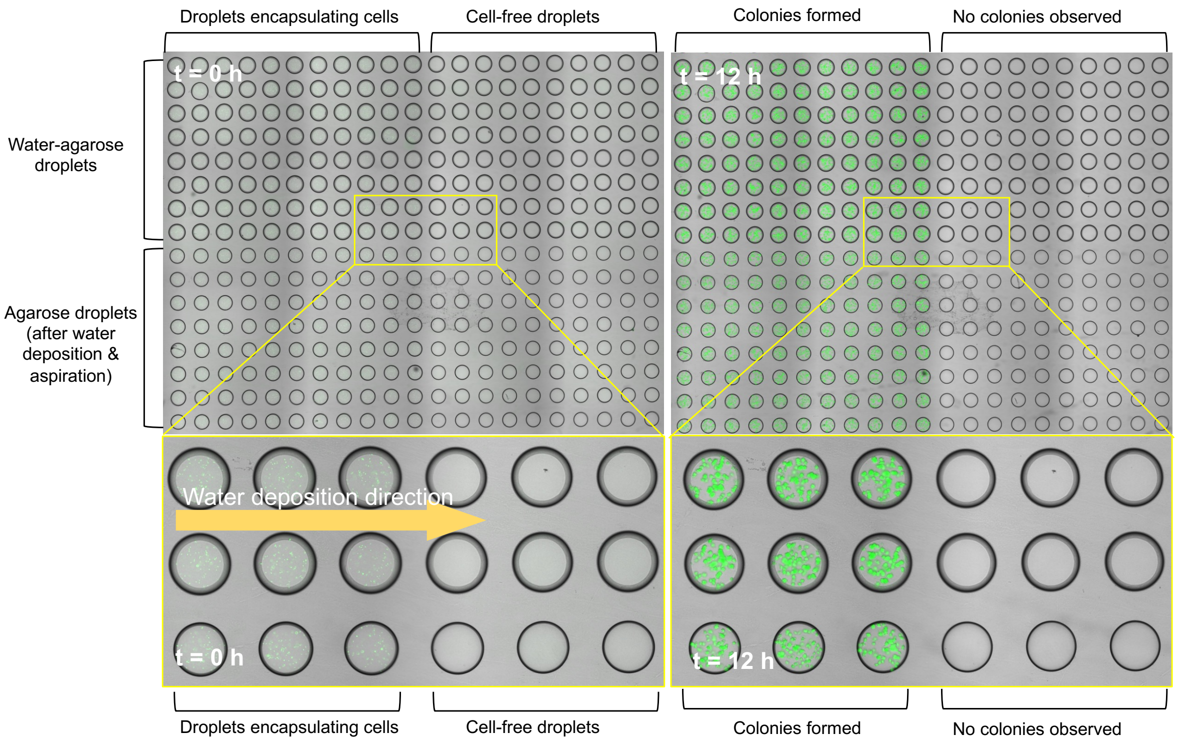

Figure S6. Microbial cross contamination test. At **t = 0 h**, agarose droplets encapsulating *E. coli* ATCC25922 cells (the **left eleven columns**) or cell-free agarose droplets (the **right ten columns**) were generated on the microarray. MHB medium was then deposited to the agarose droplets in a deposition direction of '**from left to right**' and the deposited MHB medium was aspirated on droplets at the bottom rows whereas no liquid aspiration was performed to droplets in the upper rows. **After t = 12 h cultivation**, no growth was observed in any of the cell-free agarose droplets on the right columns, suggesting no microbial cross contamination during the liquid deposition and/or aspiration.

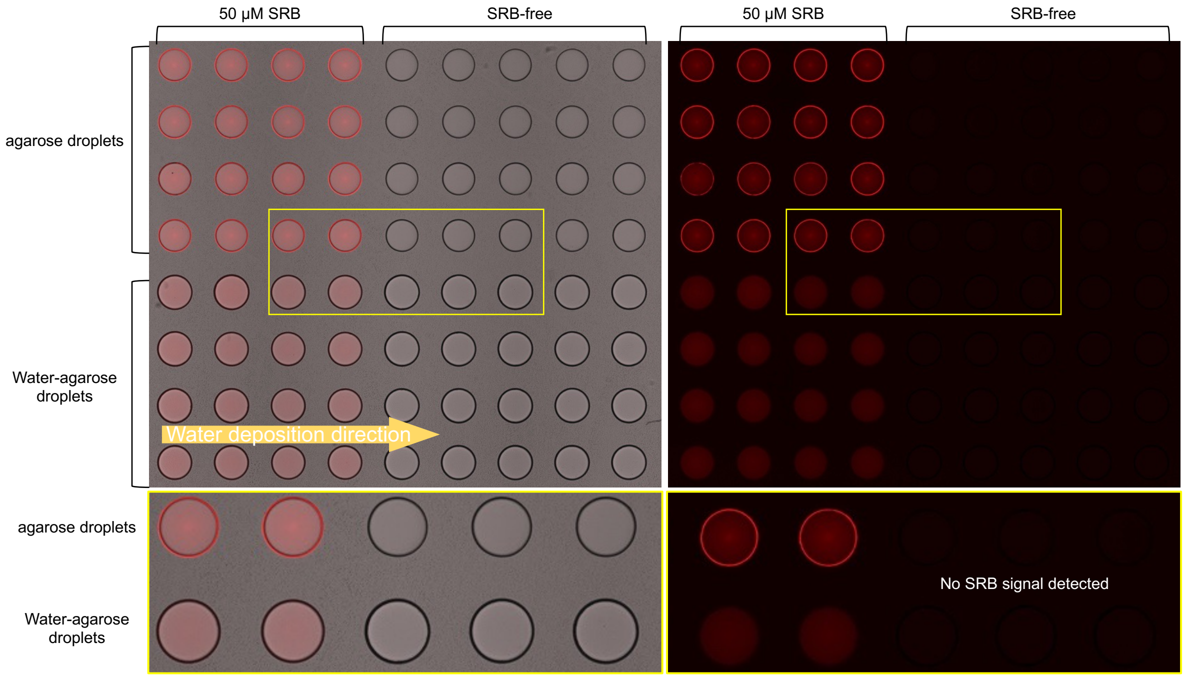

Figure S7. Chemical cross contamination test. Despite a high concentration of SRB (50 μM) was loaded in the droplet on the left side of the array, no SRB signal was detected microscopically in agarose droplets on the right columns after the liquid deposition, where the liquid deposition was performed in a direction of '**from left to right**". This indicates no (or only minor) chemical cross contamination occurred during the liquid deposition.

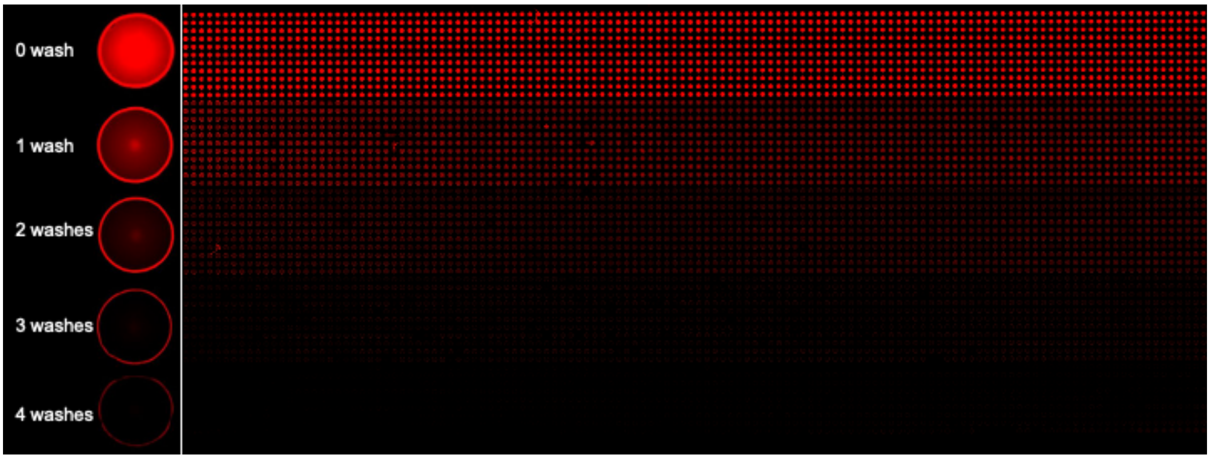

Figure S8. Reagent removal. A fluorescence micrograph of SRB-laden agarose droplets after 0, 1, 2, 3 and 4 'washing cycles'. Initially all agarose droplets on the microarray were loaded with 20-μM SRB during their formation, and then droplets at different rows were 'washed' with MHB medium for 1, 2, 3 and 4 times, respectively. SRB pre-existing in the agarose droplets were gradually 'washed' out with increasing washing cycles.

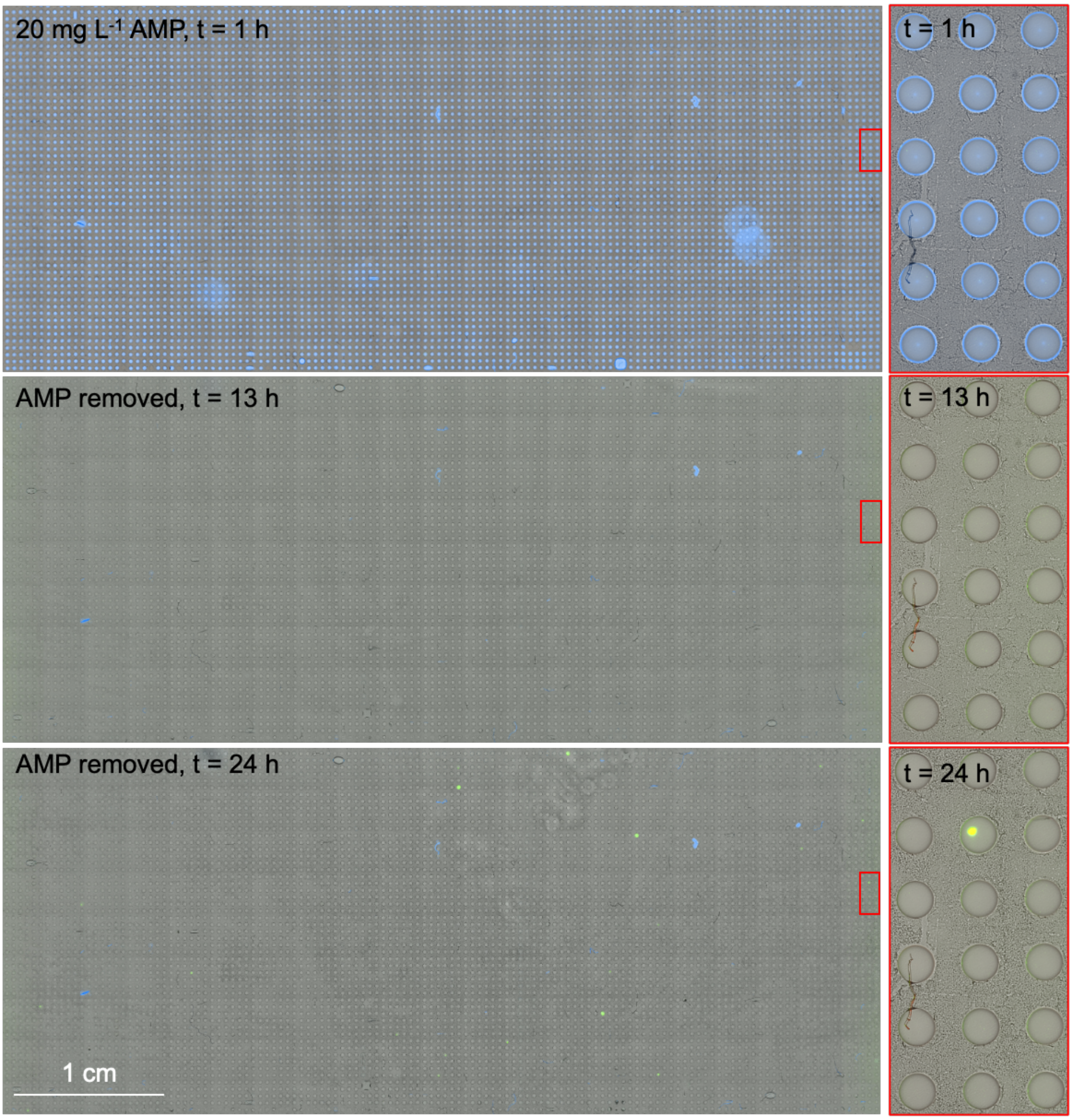

Figure S9. Transient antibiotic exposure. Micrographs of the *n* = 6784 droplets array showing bacterial colony formation within the droplets at different time points. At ***t* = 1 h**, AMP together with a blue florescent dye dextran were added to the cell-laden agarose droplets. At ***t* = 13 h**, AMP as well as the blue florescent dye dextran were removed from the agarose droplets, no colony formation was observed in the droplets. At ***t* = 24 h**, after AMP removal and then 12-h antibiotic-free incubation, some of the *E. coli* cells that survived the AMP treatment started to regrow and formed GFP-expressing colonies over time.

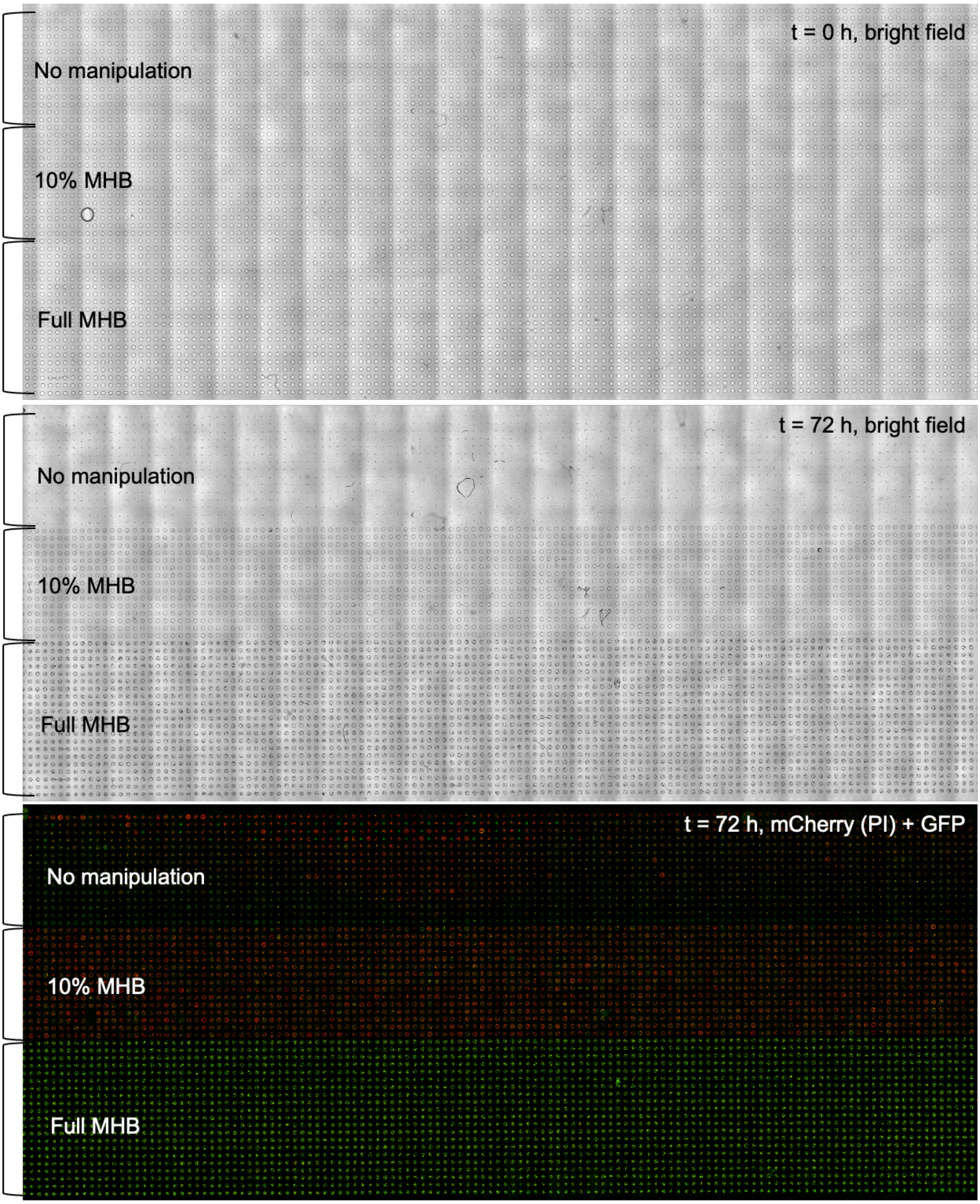

Figure S10. Medium replenishment for long term cultivation of a relatively slow-growing microorganism. Micrographs of the droplet array at $t = 0$ and 72 h. At $\boldsymbol{t = 0}$ **h**, agarose droplets (~ 2 nL per droplets) encapsulating *M. smegmatis* cells were deposited on the $n = 6784$ droplets microarray. After that, using the agarose-water two-layer droplets technique, 10% MHB and full MHB medium were repeatedly supplied to the agarose droplets located at the 17-31 and 32-52 rows of the microarray at a frequency of 12-h intervals, respectively. Whereas agarose droplets located at the 2-16 rows of the microarray served as control and no manipulation was performed on those droplets. At $\boldsymbol{t = 72}$ **h**, only in agarose droplets where full MHB medium was continuously supplied, viable and GFP-expressing (functional) colonies of *M. smegmatis*

were formed with an average colony diameter of ca. 29 µm. However, without medium replenishment or replenishment with 10% MHB, *M. smegmatis* formed significantly ($p < 0.001$) smaller colonies (d = 15-16 µm) which were observed mostly non-viable according to the dead cell stain with PI.

3. ***Calculation of droplet volume***

To calculate the volume of the agarose and aqueous layer in the two-layer droplets, below the equation calculating the volume of a spherical dome was used:

$$V = \frac{1}{6}\pi h\,(3r^2 + h^2)$$

In the equation, r and h are the base radius and height of the droplet dome, respectively. For the agarose layer, $r_{agarose}$ = 125 µm was used as after the agarose deposition, the agarose was maintained exactly within the hydrophilic spots (see below **Fig. S6**). For the volume of two-layer droplets, $r_{two\text{-}layer}$ was measured microscopically (i.e., **Fig. S6** and Table S1) after the aqueous deposition.

To measure the height of a droplet, the deposition capillary was lowered down at a precision of 1 µm untill the capillary touched the droplet and then the touching height (h_1) was recorded. After that, the capillary was further lowered down till the capillary touched the surface of the microarray plate and then a second touching height (h_2) was recorded. The height discrepancy between the two touching points was considered as the height of the droplet dome, namely $h = h_1 - h_2$. Same measurement was performed on n = 30 droplets for the agarose, and agarose-water droplets, respectively (Table S1).

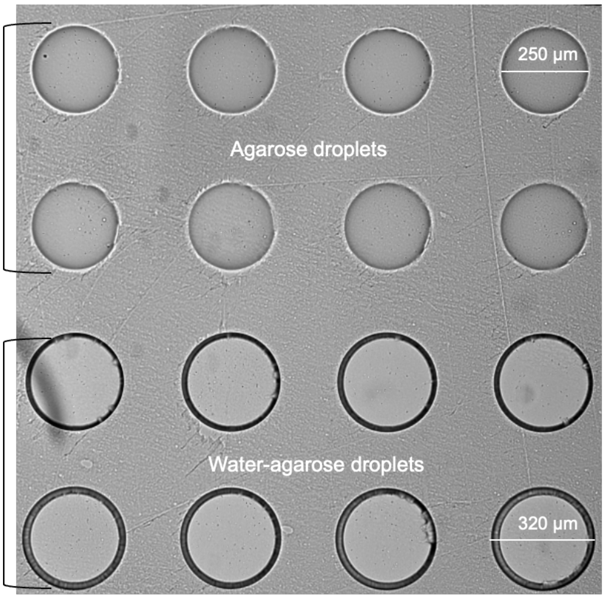

Figure S11. **A micrograph of the agarose and agarose-water two-layer droplets.** After the aqueous deposition, evidently, the droplets' base radius became bigger comparing to the base radius of the agarose droplets.

Table S1. The droplet base, height and volume of agarose and agarose-water droplets (n = 30 droplets per condition)

	Radius (r: µm)	Height (h: µm)	Droplet Volume (nL)
**Agarose droplet**	125	75 ± 2	2.0676 ± 0.03
**water-agarose droplet**	164 ± 6	115 ± 4	5.7114 ± 0.17
**Aqueous layer**			3.7382± 0.15

4. Mathematical Simulation: Diffusion in Two-Layer Droplets

To estimate the equilibrium time required for a reagent diffusing from the aqueous layer to the agarose droplet (see below **Fig. S7**), Fick's first law of diffusion was used (see below). Fick's first law of diffusion relates the diffusive flux $\boldsymbol{J}$ to the gradient of the concentration $\frac{\boldsymbol{d\varphi}}{\boldsymbol{dx}}$. It postulates that the flux goes from regions of high concentration (source) to regions of low concentration (sink), with a magnitude that is proportional to the concentration gradient as below.

$$\boldsymbol{J = -D\frac{d\varphi}{dx}} \quad \textit{(1)}$$

In equation ***(1)***,

J is the diffusion flux, it measures the amount of substance that will flow through a unit area during a unit time interval, and ***J***, expressed in kg $m^{-2} \cdot s^{-1}$.

D is the diffusion coefficient, expressed in m^2 s^{-1}.

$\frac{\boldsymbol{d\varphi}}{\boldsymbol{dx}}$ is the concentration gradient, expressed in kg m^{-4}.

$\boldsymbol{d\varphi}$ is the concentration discrepancy between the reagent source (i.e., $C_{aqueous}$) and the reagent sink (i.e., $C_{agarose}$), hence $\boldsymbol{d\varphi} = C_{aqueous} - C_{agarose}$ in our simulations,

$\boldsymbol{dx}$ is the diffusion distance between the source and the sink, i.e., $\boldsymbol{dx} = X_{aqueous} - X_{agarose}$.

To estimate the maximal equilibrium time required by fluorescein (5 mM) diffusing from the 3.7 nL aqueous layer to the 2 nL agarose droplet (**Fig. S7**), the relevant parameters were listed in **Table S2**. Using equation ***(1)***, a diffusion flux $\boldsymbol{J}_{droplet}$ can be calculated for the two-layer droplet system. Based on the ***J*** values, the time required to achieve equilibrium i.e., a final fluorescein concentration of 3.3 mM was calculated using equation ***(2)*** in **Table S2**.

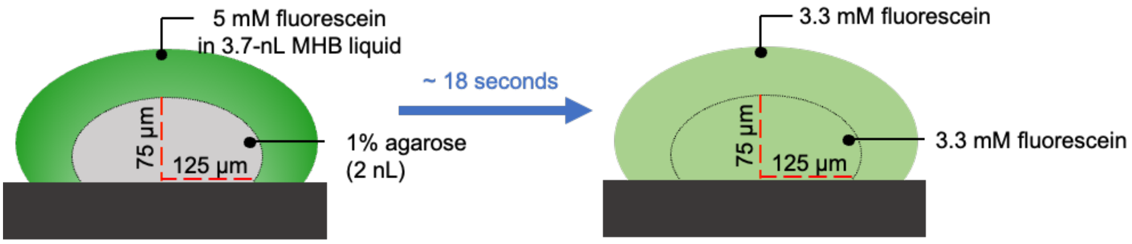

Figure S12. A sketch illustrating diffusion conditions in the two-layer droplet system.

Table S2. Parameters used to estimate the maximal equilibrium time required by fluorescein in the two-layer droplet system

**Parameters**	**Two-layer droplet system**
D (m^{-2} s^{-1}) in 1% agarose	$4.50*10^{-10}$
$C_{aqueous}$ (kg m^{-3})	1.661
$C_{agarose}$ (kg m^{-3})	1.096
$X_{aqueous}$ (m)	0.00
$X_{agarose}$ (m).	$1.25*10^{-4}$ (maximal diffusion distance)
S (m^2)	$5.89*10^{-8}$
$M = C_{agarose} \times V_{agarose}$ (kg)	$2.19*10^{-12}$
$V_{agarose}$ (m^3)	$3*10^{-12}$
J (kg m^{-2} s^{-1})	$2.03*10^{-6}$
$T = \frac{M}{S.J}$ (s) ***(2)***	18.3

Of note: D value for fluorescein is measured at room temperature (3); $C_{aqueous}$ of fluorescein (molecular weight: 332.31 g mol^{-1}) at T_0 was set to 5 mM = 1.661 kg m^3; $C_{agarose}$ of fluorescein is 3.3 mM = 1.097 kg m^3 when equilibrium is achieved. $X_{aqueous}$ was defined as zero and $X_{agarose}$ = 125 µm (radius of the spots hosting the agarose droplets); S is the surface area for diffusion in the droplet and agar plate system; M, the total mass of fluorescein required to diffuse into the agarose phase to achieve the equilibrium; J, diffusion flux calculated using equation ***(1)***; T, time required to achieve fluorescein equilibrium, calculated by dividing M with S and J.

5. References

1. Breitfeld M, Dietsche CL, Saucedo-Espinosa MA, Berlanda SF, Dittrich PS. Ultrafast Formation of Microdroplet Arrays with Chemical Gradients for Label-Free Determination of Enzymatic Reaction Kinetics. Small 2025; 21(26):2410275.
2. Surface Properties of Fibrinogen and Fibrin. Vol. 9, Journal of Protein Chemistry. 1990.
3. Shoga JS, Graham BT, Wang L, Price C. Direct Quantification of Solute Diffusivity in Agarose and Articular Cartilage Using Correlation Spectroscopy. Ann Biomed Eng. 2017;45(10):2461–74.

6. Supplementary videos

Video S1 shows the deposition of the second (aqueous) droplet on previously deposited agarose droplets.

Video S2 shows the unsuccessful deposition of the second (aqueous) droplet on previously deposited fibrin droplets.

Video S3 visualizes the aspiration of the second (aqueous) droplet from agarose droplets. See also Figure S2 for explanation.

Video S4 is produced by time-lapse microscopy, depicting colony formation in agarose droplets within 10 hours.
